# Dyslipidemic Diet-Induced Monocyte “Priming” and Dysfunction in Non-Human Primates Is Triggered by Elevated Plasma Cholesterol and Accompanied by Altered Histone Acetylation

**DOI:** 10.3389/fimmu.2017.00958

**Published:** 2017-08-22

**Authors:** John D. Short, Sina Tavakoli, Huynh Nga Nguyen, Ana Carrera, Chelbee Farnen, Laura A. Cox, Reto Asmis

**Affiliations:** ^1^Department of Pharmacology, The University of Texas Health Science Center at San Antonio, San Antonio, TX, United States; ^2^Department of Radiology, The University of Texas Health Science Center at San Antonio, San Antonio, TX, United States; ^3^Department of Biochemistry and Structural Biology, The University of Texas Health Science Center at San Antonio, San Antonio, TX, United States; ^4^Department of Molecular Medicine, The University of Texas Health Science Center at San Antonio, San Antonio, TX, United States; ^5^Department of Genetics, Texas Biomedical Research Institute, San Antonio, TX, United States; ^6^Southwest National Primate Research Center, San Antonio, TX, United States; ^7^Department of Clinical Laboratory Sciences, The University of Texas Health Science Center at San Antonio, San Antonio, TX, United States

**Keywords:** diabetes, monocytes, mitogen-activated protein kinase phosphatase 1, histone, epigenetics

## Abstract

Monocytes and the recruitment of monocyte-derived macrophages into sites of inflammation play a key role in atherogenesis and other chronic inflammatory diseases linked to cardiometabolic syndrome and obesity. Previous studies from our group have shown that metabolic stress promotes monocyte priming, i.e., enhanced adhesion and accelerated chemotaxis of monocytes in response to chemokines, both *in vitro* and in dyslipidemic LDLR^−/−^ mice. We also showed that metabolic stress-induced monocyte dysfunction is, at least to a large extent caused by the *S*-glutathionylation, inactivation, and subsequent degradation of mitogen-activated protein kinase phosphatase 1. Here, we analyzed the effects of a Western-style, dyslipidemic diet (DD), which was composed of high levels of saturated fat, cholesterol, and simple sugars, on monocyte (dys)function in non-human primates (NHPs). We found that similar to mice, a DD enhances monocyte chemotaxis in NHP within 4 weeks, occurring concordantly with the onset of hypercholesterolemia but prior to changes in triglycerides, blood glucose, monocytosis, or changes in monocyte subset composition. In addition, we identified transitory decreases in the acetylation of histone H3 at the lysine residues 18 and 23 in metabolically primed monocytes, and we found that monocyte priming was correlated with the acetylation of histone H3 at lysine 27 after an 8-week DD regimen. Our data show that metabolic stress promotes monocyte priming and hyper-chemotactic responses in NHP. The histone modifications accompanying monocyte priming in primates suggest a reprogramming of the epigenetic landscape, which may lead to dysregulated responses and functionalities in macrophages derived from primed monocytes that are recruited to sites of inflammation.

## Introduction

Cardiometabolic syndrome (CMS) is a combination of metabolic disorders or risk factors, including abdominal obesity, hypertension, dyslipidemia [elevated low-density lipoprotein (LDL)-cholesterol, elevated triglycerides and low levels of circulating high-density lipoproteins], and hyperglycemia (1, 2). CMS affects more than one-fifth of US adults over the age of 20 and is becoming a global burden (1, 2). Furthermore, CMS increases the risk of various diseases, including type 2 diabetes (T2D), cardiovascular disease, heart failure, kidney disease, and certain types of cancer (2, 3). More recently, systemic inflammation has been associated with obesity, CMS, T2D, and cardiovascular disease (4). For example, there is a vast infiltration of blood monocyte-derived macrophages (MDMs) into the adipose tissue of obese mice and of obese patients (5, 6), and MDM recruitment is a rate-limiting event that drives atherosclerosis related to CMS (7, 8).

Several genetic and dietary mouse models have been used to study CMS, and these models have been classified according to which aspects of CMS they most closely mimic in human pathophysiology, including (i) obesity and insulin resistance, (ii) hypertension or obesity with hypertension, (iii) lipodystrophy, and (iv) hyperlipidemia (9). For example, the low-density lipoprotein receptor-deficient (LDLR^−/−^) mouse, which is a model of hyperlipidemia (9), develops hypercholesterolemia with elevated LDL (10). In response to a “Western-type diet” with high-fat content (42% of calorie content) and high cholesterol levels (0.15%), these mice develop obesity, severe hyperlipidemia, insulin resistance, and atherosclerosis (11, 12). Previous studies from our group have shown that a dyslipidemic, atherogenic diet (i.e., high-fat and high cholesterol) or diabetic conditions (high-fat diet + streptozotocin) also enhances monocyte chemotaxis and the recruitment rate of MDMs in LDLR^−/−^ mice in response to chemokines, thereby contributing to the enhanced macrophage content in atherosclerotic lesions (13, 14). Importantly, the degree of monocyte priming was a strong predictor of the rate of atherogenesis (13). This metabolic stress-enhanced chemotactic activity of blood monocytes, which we termed “metabolic priming” of monocytes (13, 15), and the enhanced recruitment of MDMs to sites of inflammation can also be recapitulated *in vitro* by stressing monocytes with hyperglycemic and hyperlipidemic conditions (15–18). More recently, we showed that metabolic priming of monocytes, both *in vitro* and *in vivo*, is mediated, at least in part, by the *S*-glutathionylation, inactivation, and degradation of mitogen-activated protein kinase phosphatase 1 (MKP-1) (17). Importantly, MKP-1 activity is dramatically reduced in blood monocytes of diabetic mice (17), and hematopoietic MKP-1 deficiency in LDLR^−/−^ mice promotes atherosclerosis (19). We also showed that loss of MKP-1 activity in response to metabolic priming leads to increased p38 mitogen-activated protein kinase (p38 MAPK) and extracellular signal-regulated kinase (ERK) signaling in MCP-1-stimulated THP-1 monocytes (17). Lastly, our lab demonstrated that overexpression of glutaredoxin 1 (Grx1), an enzyme that reverses protein *S*-glutathionylation (7), blocks the effects of metabolic stress on monocyte chemotaxis and protects MKP-1 from inactivation (15, 17). Together, these findings strongly suggest that metabolic stress-induced monocyte priming and dysfunction, which is mediated by MKP-1 *S*-glutathionylation and inactivation, contributes to and accelerates atherogenesis.

The baboon has served as one of the primary primate models for understanding obesity and CMS (20). Baboons, like humans and many other non-human primates (NHPs), are known to develop metabolic abnormalities, such as obesity and dyslipidemia, as they age (20). In addition, high-fat diets and diets rich in simple sugars have long been known to cause an increase in atherosclerotic lesions in these animals (21–23). More recently, baboons fed a diet rich in saturated fats and simple sugars along with a liquid source of simple sugars were shown to have increased adiposity and dyslipidemia after only 8 weeks of this dietary regimen (24). These baboons also displayed elevated adipokine concentrations and increased levels of glycated HbA1c (24), recapitulating several features of human CMS.

The primary objective of this study was to assess whether blood monocytes are metabolically primed early during the progression of CMS in an NHP model, since the metabolic priming of blood monocytes has thus far only been demonstrated in mouse models of CMS. Furthermore, this study aims to identify novel biomarkers, including possible epigenetic changes, that are associated with metabolic priming of blood monocytes in NHP in response to a dyslipidemic diet (DD).

## Materials and Methods

### Animals and Diets

Thirteen male baboons (*Papio hamadryas* sp.) between the ages of 5 and 7 years were studied. All baboons were from the Southwest National Primate Research Center colony and had no prior significant medical history. Standard health assessments were performed on all animals by a veterinarian prior to initiating the study. All animals were group-housed in outdoor enclosures according to established National Research Council guidelines, and all procedures were approved by the Institutional Animal Care and Use Committee of the Texas Biomedical Research Institute (San Antonio, TX, USA). Animals were fed a maintenance diet (MD: 18% protein, 13% fat, and 69% carbohydrates) from LabDiet^®^ (Monkey Diet #5038) or a dyslipidemia-inducing diet enriched in monosaccharides and saturated fatty acids (DD: 7% lard, 4% Crisco, 4% coconut oil, 0.15% cholesterol wt/wt, and 10.5% high fructose corn syrup wt/wt) for 8 weeks (24).

### Blood Analysis

Fasting plasma concentrations of glucose, total cholesterol, LDL + very low-density lipoprotein (VLDL) cholesterol (LDL + VLDL), high-density lipoproteincholesterol (HDL), and triglycerides were measured using an Unicel DxC 600 Synchron Clinical System (Beckman Coulter). The normal range of total cholesterol was anticipated to be 53–146 mg/dL, and the normal HDL levels in these animals have been reported previously to be approximately 62 mg/dL (25). LDL + VLDL was calculated by subtracting HDL from total cholesterol levels for each animal. Complete blood counts were determined using an Unicel DxH 800 Coulter^®^ Cellular Analysis System (Beckman Coulter).

### Monocyte Purification and Subset Analysis

Whole blood (3.3 mL/kg body weight, up to 75 mL) was collected immediately prior to initiating the diets (week 0) and at weeks 4 and 8 of the dietary regimens. Blood was immediately diluted at a 1:1 ratio with PBS containing 2% fetal bovine serum (PBS-2% FBS). Diluted blood was then separated in SepMate™ tubes using Lymphoprep™ density gradient centrifugation medium (Stemcell Technologies) according to the manufacturer’s protocol, and washed twice with PBS-2% FBS. Cells were then lysed twice at room temperature (RT) for 10 min each with red blood cell lysis solution (155 mM NH_4_Cl, 14 mM NaHCO_3_, 0.1 mM EDTA, pH = 7.3). After centrifugation (400 × *g* for 10 min), cells were placed in separation buffer (PBS, 0.5% BSA, 2 mM EDTA) and magnetically separated using NHP CD14 Microbeads (MACS Miltenyi Biotec) according to the manufacturer’s protocol.

After red blood cell lysis of whole blood, purified monocytes or total blood leukocytes were stained with the following antibodies: Brilliant violet 421-conjugated anti-human cluster of differentiation 10 (BioLegend, clone HI10a); phycoerythrin-conjugated anti-human CD11b (eBioscience, clone ICRF44); allophycocyanin-conjugated anti-human CD14 (MACS Miltenyi Biotec, clone TÜK4); and AlexaFluor 488-conjugated anti-human CD16 (BioLegend, clone 3G8). Labeled cells were then fixed with PBS containing 2% paraformaldehyde (PBS-2% PFA) and analyzed by flow cytometry using a BD LSR-II instrument, and data were analyzed with FACSDiva (BD Biosciences) and FloJo v.10 software (FloJo, LLC).

### *Ex Vivo* Monocyte Chemotaxis

Immediately after purification, baboon blood monocytes were resuspended at a concentration of 7.5 × 10^5^ cells/mL in “complete growth medium” (a 1:1 mixture of Hyclone RPMI 1640 and glucose-free RPMI 1640 from Cellgro) containing 5% human serum (Valley Biomed), 5.5 mM glucose, 2 mM glutamax (Cellgro), 1 mM sodium pyruvate (Cellgro), and 100 IU/mL penicillin/streptomycin (Cellgro). Cells were then loaded into the upper wells of a 48-well modified Boyden chamber (NeuroProbe, Inc.) in which the lower wells contained either vehicle or 2 nM recombinant mouse C-C motif chemokine ligand 2 (CCL2: R&D Systems). A 5 µm polyvinyl pyrrolidone-free polycarbonate filter was layered between the chambers, and the unit was incubated for 2.5 h in a 37°C incubator with 5% CO_2_. The filter was then washed to remove cells from the upper side, and the lower side was fixed with 100% methanol for 60 s and then allowed to air dry. Fixed cells were then stained with propidium iodide (1 µM; Sigma) for 30 min at RT and then washed with distilled H_2_O for 10 min. Cells were then imaged and quantified using a Kodak Image Station 4000MM (excitation: 535 nm, emission: 600 nm) and Carestream Molecular Imaging software (Nguyen et al., submitted^1^.

To metabolically “prime” purified baboon blood monocytes, cells were resuspended at 0.4 × 10^6^ cells/mL and cultured in Teflon bags under non-adherent conditions in a 37°C incubator with 5% CO_2_ (26). Cells (2 mL) were cultured overnight in complete growth media as described above or cultured in complete growth media supplemented with 20 mM glucose and 100 µg/mL freshly isolated human LDL. After 20 h, cells were resuspended at a concentration of 4.0 × 10^5^ cells/mL in complete growth media, and chemotaxis assays were performed as described above. For all experiments, cell viability was assessed by Trypan Blue exclusion (0.2% Trypan Blue in saline, Sigma) using a Cellometer Vision instrument and software (Nexcelom Bioscience).

### MKP-1 Activity Assay

Mitogen-activated protein kinase phosphatase 1 activity assays were performed with a modified commercially available Malachite green (MG)-based protein tyrosine phosphatase assay, as described previously (17, 18). Briefly, purified blood monocytes were washed with Tris-buffered saline and lysed in a mild lysis buffer (20 mM Tris–HCl, 150 mM NaCl, 1% NP-40, pH 7.5). Lysates (2 µg protein) were incubated with a phosphotyrosine peptide (200 µM; EMD Millipore) at 30°C for 10 min in the absence or presence of 40-µM sanguinarine chloride (SaCl; R&D Systems). The reaction was then stopped and MG blue solution was added for an additional 10 min at RT. Absorbance (620 nm) was quantified using a VersaMax plate reader (Molecular Devices) with KH_2_PO_4_ as a standard to measure both total phosphate release from the peptide and SaCl-dependent phosphate cleavage.

### Western Blotting

Purified blood monocytes were lysed using 0.5% SDS lysis buffer (50 mM Tris–HCl, 0.5% SDS, pH 8.0) containing 1× Pierce™ Protease and Phosphatase Inhibitor (ThermoFisher Scientific). Lysate quantification and normalization, as well as Western blotting, was performed as described previously (27). All primary antibodies except anti-Grx1 (R&D Systems) were from Cell Signaling, Inc., and secondary antibodies conjugated to horseradish peroxidase were from Santa Cruz Biotechnology or from Jackson ImmunoResearch Laboratories, Inc.

### Statistical Analyses

All data were analyzed using SigmaPlot 12.0 software (SigmaStat, Inc.). Baboon characteristics, e.g., body weight, blood lipids, and CBC analysis, as well as cellular and biochemical assays were tested by one-way repeated measures ANOVA with Holm–Sidak *post hoc* test. The *in vitro* monocyte chemotaxis assay data were analyzed by a Student’s *t*-test. All data were tested for normality using a Shapiro–Wilk test, and results were considered significant at the *P* < 0.05. Linear regression analyses were also performed using SigmaPlot 12.0 software.

## Results

### Diet-Induced Early-Stage Hypercholesterolemia in Baboons

**Figure 1:**
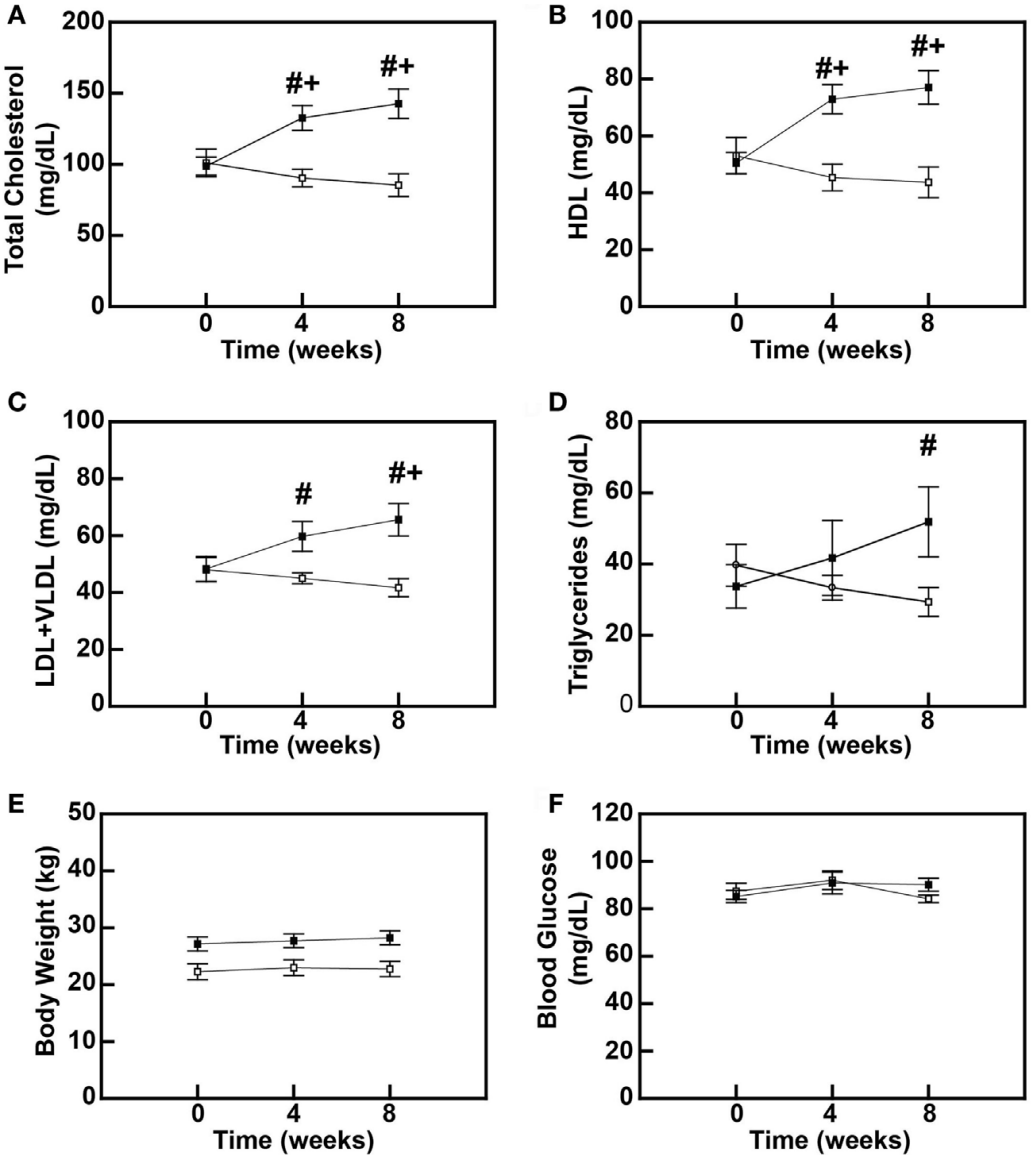
High-fat diet feeding causes dyslipidemia in baboons. Serum cholesterol levels **(A)**, high-density lipoprotein cholesterol (HDL) levels **(B)**, low-density lipoprotein (LDL) + very low-density lipoprotein (VLDL) levels **(C)**, serum triglyceride levels **(D)**, body weight **(E)**, and blood glucose levels **(F)** of male baboons fed a maintenance diet (MD = □; *n* = 6) or a high-fat dyslipidemic diet (DD = ■; *n* = 7) for up to 8 weeks. All data points represent the mean ± SEM. ^#^*P* < 0.05 when comparing diets between groups at same time point; ^+^*P* < 0.05 when comparing within the same dietary group to its corresponding pre-diet (0-week) measure.

To determine the early-stage effects of a diet rich in saturated fats, cholesterol, and simple sugars on blood lipids in baboons, male baboons between 5 and 6 years of age were fed either an MD or a DD for up to 8 weeks. Total plasma cholesterol levels were increased by 31% (*P* = 0.006) and 41% (*P* < 0.001) at 4 and 8 weeks, respectively (Figure 1A), and this hypercholesterolemia was due to increases in both plasma HDL and LDL + VLDL levels (Figures 1B,C). Plasma HDL levels increased 44.6% (*P* = 0.001) and 52.7% (*P* < 0.001) at 4 and 8 weeks, respectively (Figure 1B), whereas LDL + VLDL levels increased 23.7% (*P* = 0.131) after 4 weeks and 35.8% (*P* = 0.008) after 8 weeks (Figure 1C). Plasma triglyceride levels were similar to LDL + VLDL in that they increased throughout the diet feeding period, but differences between the DD and the MD groups only reached statistical significance after 8 weeks (53.4%; *P* = 0.009) (Figure 1D). Furthermore, these changes in blood lipids preceded any changes in baboon weights (Figure 1E) and blood glucose levels (Figure 1F), which were not significantly different between MD-fed and DD-fed baboons over the course of this 8-week study. These data indicate that, in primates, a diet rich in saturated fats, cholesterol, and simple sugars causes hypercholesterolemia and dyslipidemia within 4 weeks.

### Early-Stage Hypercholesterolemia Is Correlated with Hyper-Responsiveness of Baboon Monocytes to MCP-1

**Figure 2:**
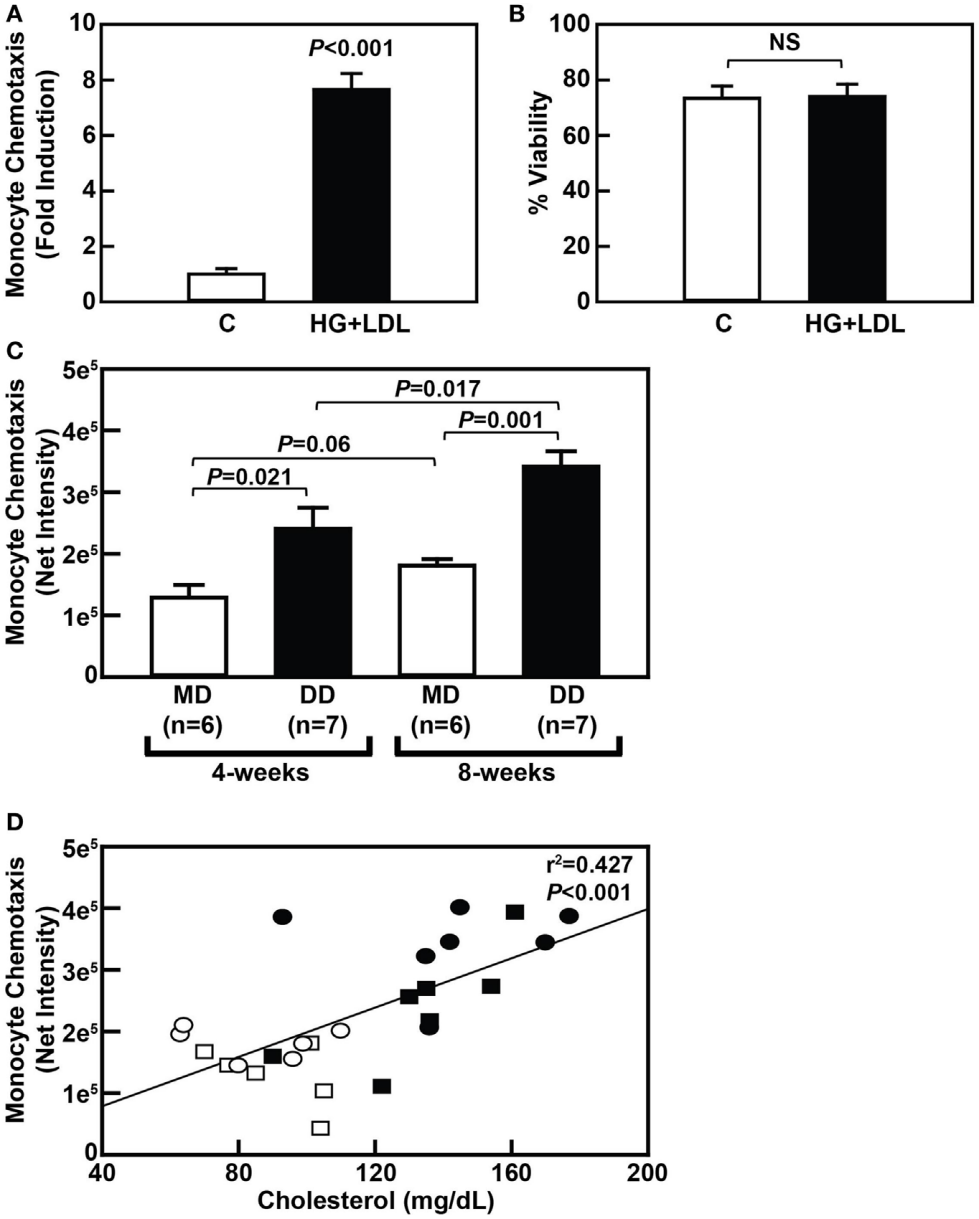
Dyslipidemic diet (DD)-induced metabolic priming of baboon monocytes is correlated with hypercholesterolemia. **(A)** MCP-1-dependent chemotaxis of purified baboon monocytes that were cultured overnight in complete RPMI culture media with 5 mM glucose (C) or media containing an additional 20 mM glucose and 100 µg/mL low-density lipoprotein (LDL) (HG + LDL). Chemotaxis data (mean ± SEM) were normalized to the control group at each time point and is expressed as “Fold Induction.” **(B)** Cell viability of purified baboon monocytes described in panel **A** (mean ± SEM). NS, not statistically significant. **(C)** MCP-1-dependent chemotaxis of purified monocytes from baboons that were fed an maintenance diet (MD) (□) or a DD (■) for either 4 or 8 weeks (mean ± SEM). **(D)** Linear regression analysis assessing the relationship between serum cholesterol levels and monocyte chemotaxis in baboons fed an MD for 4 weeks (□) or 8 weeks (⚪), or a DD for 4 weeks (■) or 8 weeks (⚫). Exact *P*-values are shown for the chemotaxis data **(A,C)**, and the exact *r*^2^ and *P*-values for the correlation analysis are shown **(D)**.

To determine if metabolic stress promotes NHP monocyte hyper-responsiveness to chemokines similarly to that seen in rodents, we first purified primary monocytes from freshly drawn baboon blood and exposed the cells to metabolic stress, i.e., 20 mM glucose plus 100 µg/mL freshly isolated human LDL (HG + LDL). Our purification strategy resulted in a greater than 90% pure monocyte population (Figure S1 in Supplementary Material). Treatment of purified baboon monocytes with HG + LDL resulted in an approximately sevenfold increase in MCP-1-mediated chemotaxis when compared with monocytes cultured in physiological glucose levels, i.e., 5 mM glucose (Figure 2A), indicating that primary NHP blood monocytes, like murine and human blood monocytes, are capable of being metabolically primed. Treatment of these cells with HG + LDL did not affect monocyte viability (Figure 2B).

To determine whether a DD causes hyper-responsiveness of blood monocytes in NHP *in vivo*, MCP-1-mediated recruitment of purified blood monocytes isolated from DD-fed baboons was compared with purified monocytes from MD-fed baboons at both the 4- and 8-week time points. Indeed, MCP-1-mediated chemotaxis of purified monocytes from baboons fed a DD was increased 1.86-fold (*P* = 0.022) and 1.88-fold (*P* = 0.001) when compared with purified monocytes from MD-fed animals at the 4- and 8-week time points, respectively (Figure 2C). Furthermore, MCP-1-mediated chemotaxis of purified monocytes from baboons fed a DD for 8 weeks was significantly higher (1.42-fold; *P* = 0.017) when compared with monocytes from the same baboons fed a DD for 4 weeks (Figure 2C). These data indicate that monocytes from DD-fed baboons are primed as early as 4 weeks after initiating a DD and continue to have increased monocyte chemotaxis rates over the course of 8 weeks. Importantly, the onset of monocyte priming coincided with an increase in total plasma cholesterol levels (compare Figures 2C and 1A), and there was a highly significant correlation (*P* < 0.001) between plasma cholesterol levels and monocyte chemotaxis (Figure 2D). These data strongly suggest that hypercholesterolemia alone is sufficient to promote monocyte priming in NHP *in vivo*.

### Early-Stage Hypercholesterolemia Is Correlated with Neutrophilia in NHP

**Figure 3:**
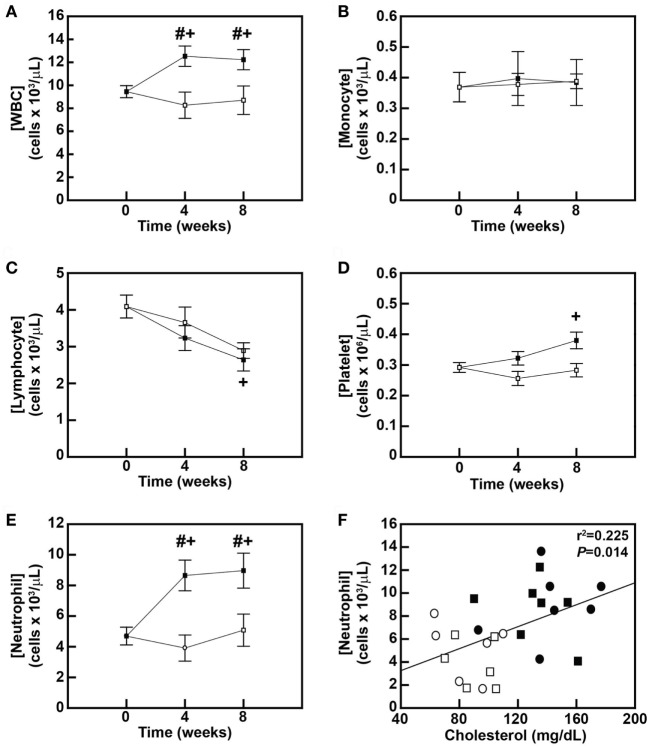
Dyslipidemic diet (DD)-induced neutrophilic leukocytosis is correlated with hypercholesterolemia in baboons. Concentrations (mean ± SEM) of blood leukocytes **(A)**, monocytes **(B)**, lymphocytes **(C)**, platelets **(D)**, and neutrophils **(E)** in male baboons fed an maintenance diet (MD) (□; *n* = 6) or a DD (■; *n* = 7) for up to 8 weeks. ^#^*P* < 0.05 when comparing between groups at same time point; ^+^*P* < 0.05 when comparing within the same group with 0-week time point. **(F)** Linear regression analysis examining the relationship between serum cholesterol levels and blood neutrophil concentrations in baboons fed an MD for 4 weeks (□) or 8 weeks (⚪), or a DD for 4 weeks (■) or 8 weeks (⚫). The exact *r*^2^ and *P-*value for the correlation analysis are shown.

To further assess the effects of the DD on monocytes and other blood leukocytes and to correlate dyslipidemia with these effects, leukocyte counts obtained by complete blood count analyses were first compared between diet groups after 4 and 8 weeks of diet feeding. The DD stimulated a 32.6% increase (*P* = 0.027) in blood leukocyte counts within 4 weeks of initiating the diet (Figure 3A), and this significant increase in leukocytosis was maintained for the remainder of the 8-week study (*P* = 0.039; Figure 3A). No changes in white blood cells were observed in the MD-fed animals over the 8-week feeding period (Figure 3A). Surprisingly, there was no change in blood monocyte levels over the 8-week feeding period in either group (Figure 3B). We also analyzed whether the DD altered monocyte subset composition in baboons (Figure S1A in Supplementary Material) since both CD14^lo^CD16^hi^ and CD14^hi^CD16^hi^ monocyte subsets are increased in obese patients (28). However, the monocyte subset composition was also not altered in DD-fed baboons over the course of the 8-week feeding period (Figure S1A and Table S1 in Supplementary Material). Blood lymphocyte counts were decreased by 29.3% in the MD group (*P* = 0.074) and 35.5% (*P* = 0.017) in the DD group at the 8-week time point (Figure 3C); however, there was no statistically significant difference in the lymphocyte count between the two dietary groups at the 8-week time point (*P* = 0.674) (Figure 3C). The DD-fed group also displayed a 30.1% increase (*P* = 0.044) in platelet counts in response to 8 weeks of DD feeding (Figure 3D); however, the difference in platelet concentrations between DD-fed baboons and MD-fed baboons also did not reach statistical significance (*P* = 0.163; Figure 3D).

Leukocytosis in DD-fed baboons (Figure 3A) was primarily due to an increase in neutrophils, which were increased by 84.4% (*P* = 0.001) after 4 weeks and by 91.2% (*P* < 0.001) after 8 weeks (Figure 3E). Furthermore, there was a significant correlation (*P* = 0.014) between plasma cholesterol levels and blood neutrophil levels in baboons (Figure 3F). These data suggest that a DD induces early changes in blood neutrophils, which like monocyte priming appear to be triggered by rising blood cholesterol levels.

### Hypercholesterolemia Is Correlated with Loss of Monocytic MKP-1 Activity

**Figure 4:**
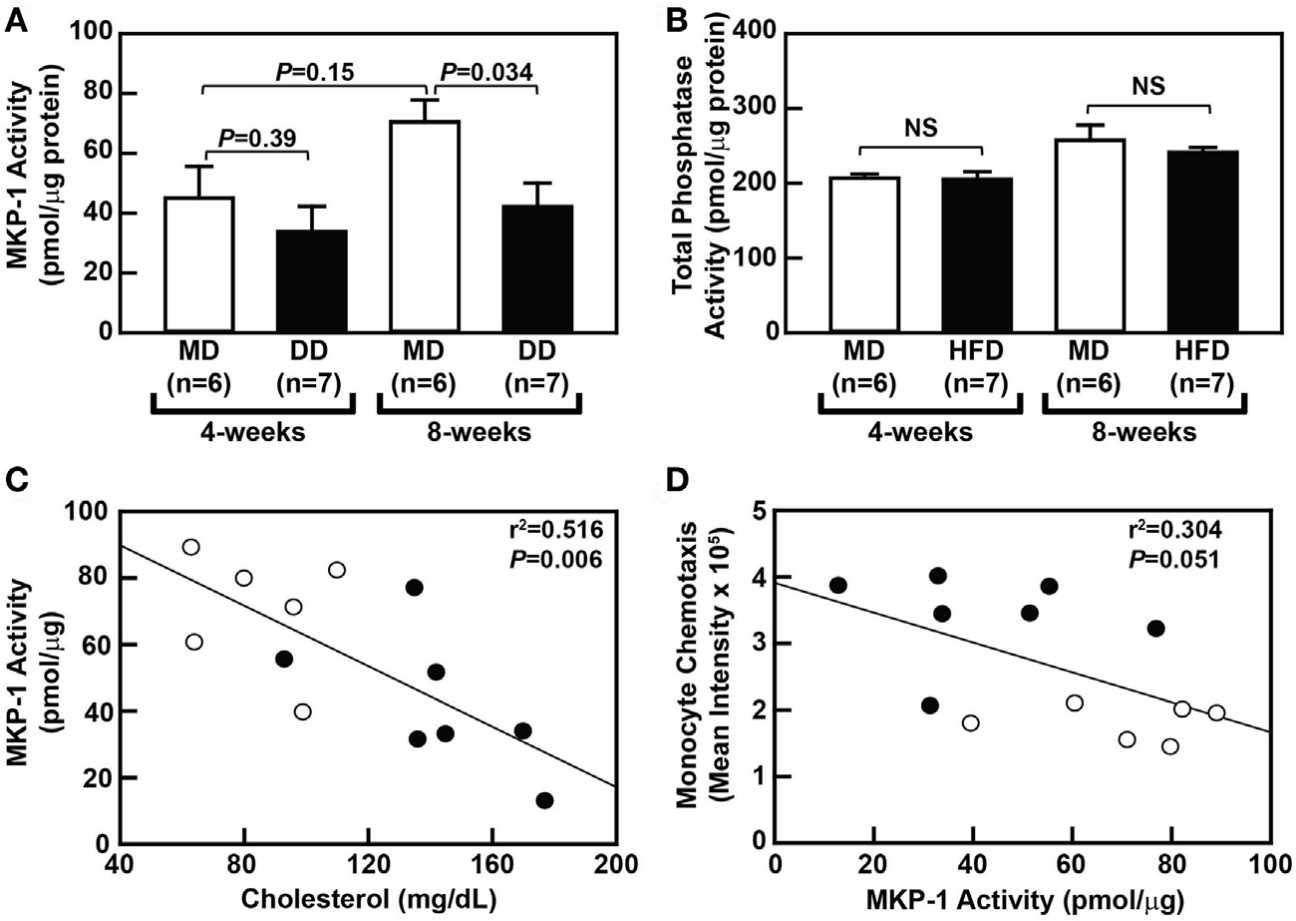
Dyslipidemic diet (DD) feeding leads to reduced monocytic mitogen-activated protein kinase phosphatase 1 (MKP-1) activity in baboons. **(A)** Sanguinarine-dependent protein tyrosine phosphatase (PTP) phosphate release (mean ± SEM) from purified baboon monocyte lysates. Lysates were generated using purified monocytes from baboons that were fed an maintenance diet (MD) (□) or a DD (■) for either 4 or 8 weeks. **(B)** Total PTP phosphate release (mean ± SEM) from purified baboon monocyte lysates described in panel **A**. Phosphate release was normalized to protein concentration, and the exact *P*-values are shown for each time point **(A)**. NS, not statistically significant. **(C)** Linear regression analysis assessing the relationship between serum cholesterol levels and MKP-1 activity in monocytes from baboons fed an MD (⚪) or a DD for 8 weeks (⚫). **(D)** Linear regression analysis examining the relationship between MKP-1 activity and chemotaxis of monocytes from baboons fed an MD (⚪) or a DD (⚫) for 8 weeks. The exact *r*^2^ values and *P*-values for the correlation analyses are shown **(C,D)**.

To determine whether metabolic priming of baboon monocytes is mediated by a loss of MKP-1 activity, we compared sanguinarine-dependent monocytic phosphatase activity, a measure of MKP-1 activity, in purified monocytes from MD-fed and DD-fed baboons. Interestingly, an MD regimen led to a slight increase in MKP-1 activity over the course the 8-week dietary regimen (Figure 4A), although this increase was not statistically significant (*P* = 0.147). When comparing monocytic MKP-1 activity in DD-fed baboons with MD-fed baboons, MKP-1 activity was reduced by 25% (*P* = 0.39) and 40% (*P* = 0.034) at 4 and 8 weeks, respectively (Figure 4A). Importantly, total phosphatase activity was not significantly altered between the groups (Figure 4B). There was also a statistically significant correlation (*P* = 0.006) between plasma cholesterol levels and monocytic MKP-1 activity in baboons at the 8-week time point (Figure 4C); however, plasma cholesterol levels and monocytic MKP-1 activity were not significantly correlated (*P* = 0.97) at the 4-week time point (data not shown). Furthermore, monocytic MKP-1 activity and monocyte chemotaxis were not significantly correlated at the 8-week time point (*P* = 0.051; Figure 4D). Taken together, these data suggest that loss of monocytic MKP-1 activity appears to be induced by increasing plasma cholesterol levels, but only partially accounts for metabolic priming of monocytes, with additional factors contributing to aberrant monocyte chemotaxis associated with hypercholesterolemia in NHPs.

### A DD Does Not Affect Grx1 Protein Levels or Baseline ERK Signaling in Baboon Monocytes

**Figure 5:**
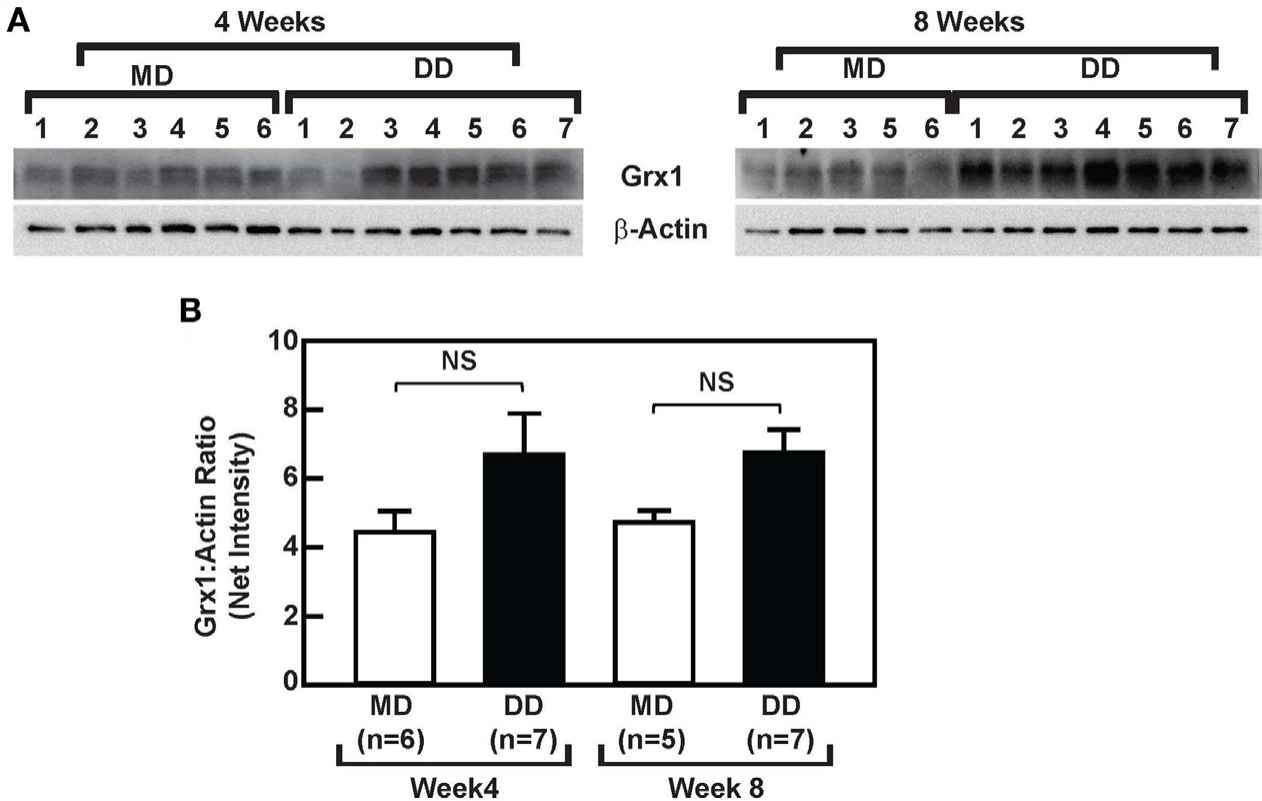
Dyslipidemic diet (DD) feeding does not affect glutaredoxin 1 (Grx1) expression in baboon monocytes. **(A)** Western blot analysis of Grx1 and β-actin using lysates from monocytes, which were purified from baboons that were fed an maintenance diet (MD) or a DD for either 4 weeks (left panels) or 8 weeks (right panels). **(B)** The density (mean net intensity ± SEM) of each protein at each time point was calculated, and the ratio of Grx1:β-actin is shown. NS, not statistically significant.

To determine whether the loss of MKP-1 activity in response to metabolic priming increased p38 MAPK and ERK signaling in baboon monocytes and to assess the effects of metabolic stress on Grx1 protein levels, we compared ERK and p38 MAPK signaling in monocytes from DD-fed baboons with monocytes from the MD-fed group, and we assessed whether a DD-mediated reduction in Grx1 levels accounted for the reduced MKP-1 activity in monocytes from DD-fed baboons, at least at the 8-week time point (Figure 4A). Phosphorylated ERK (p-ERK^T202/Y204^) and phosphorylated p38 (p-p38^T180/Y182^) levels in purified monocytes from DD-fed baboons were not significantly altered when compared with those from MD-fed animals (Figure S2 in Supplementary Material). In addition, monocytic Grx1 protein levels in the DD-fed group were not decreased but instead slightly increased when compared with Grx1 levels in monocytes from the MD-fed group (Figure 5); however, differences in Grx1 protein levels in monocytes between the two groups were not statistically significant (Figure 5B). These data indicate that baseline ERK and p38 MAPK signaling are not affected by suppressed MKP-1 activity in monocytes from DD-fed baboons. Furthermore, these data suggest that changes in Grx1 expression levels do not account for the effects of a DD on MKP-1 activity in baboon monocytes.

### Dynamic Changes in Monocytic Histone H3 Acetylation in MD- and DD-Fed Baboons

**Figure 6:**
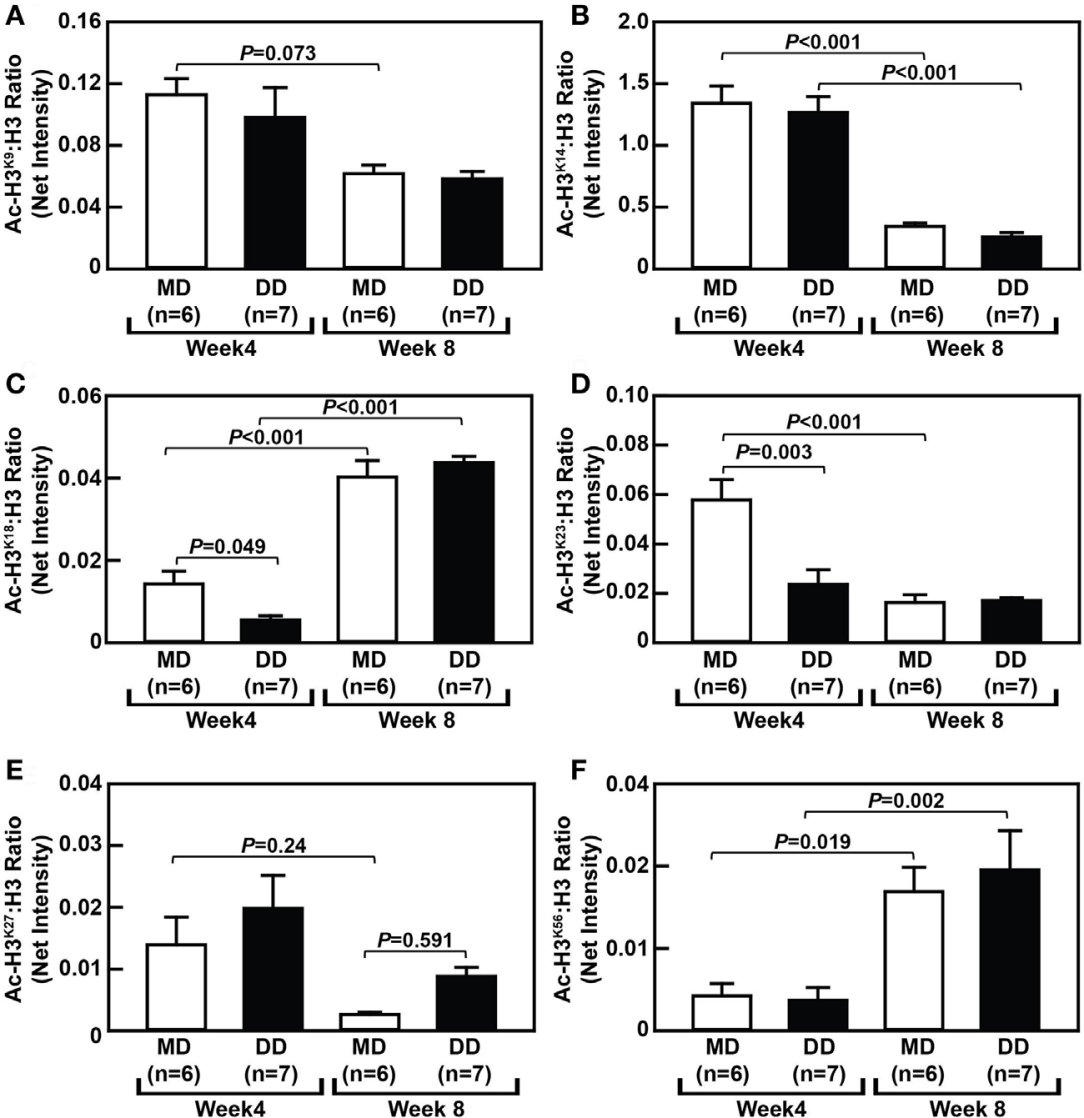
Dyslipidemic diet (DD) feeding leads to reduced histone H3 acetylation at lysine 18 and 23 in baboon monocytes. The densitometric ratio (mean ± SEM) of Ac-H3^K9^: H3 **(A)**, Ac-H3^K14^: H3 **(B)**, Ac-H3^K18^: H3 **(C)**, Ac-H3^K23^: H3 **(D)**, Ac-H3^K27^: H3 **(E)**, and Ac-H3^K56^: H3 **(F)**, which were calculated using Western blot images shown in Figure S3 in Supplementary Material. Exact *P*-values are indicated.

To assess changes in histone acetylation in metabolically primed NHP monocytes, we analyzed the acetylation status of histone H3 (H3) at several lysine residues within monocytes from MD-fed and DD-fed baboons. Surprisingly, we found that H3 acetylation at certain lysine residues was significantly altered in monocytes from baboons that were switched from their regular chow to an MD for 4 and 8 weeks. For example, acetylated H3 at lysine 14 (Ac-H3^K14^) and Ac-H3^K23^ were significantly decreased in monocytes from MD-fed baboons (*P* < 0.001) when comparing cells at the 8-week time point with the 4-week time point (Figures 6B,D), while Ac-H3^K18^ (*P* < 0.001) and Ac-H3^K56^ (*P* = 0.019) were significantly increased in monocytes from the MD-fed group at 8 weeks (Figures 6C,F). Although Ac-H3^K9^ and Ac-H3^27^ were decreased in monocytes from MD-fed baboons when comparing cells at the 8-week time point with the 4-week time point, these differences were not statistically significant (Figures 6A,E). These data suggest that the MD regimen has selective effects on H3 acetylation at various residues over the course of this 8-week study.

Although the MD had a selective effect on H3 acetylation during this 8-week study, monocytes from NHPs on a DD showed major temporal changes in histone H3 acetylation at several residues (Figure 6). Acetylation of lysine 18 (Ac-H3^K18^) was reduced by 39% (*P* = 0.049) and Ac-H3^K23^ was reduced by 41% (*P* = 0.003) in monocytes from DD-fed baboons at the 4-week time point when compared with MD-fed baboons (Figures 6C,D). However, acetylation at both of these residues had recovered after 8 weeks and there was no longer significantly different than Ac-H3^K18^ or Ac-H3^K23^ in monocytes from DD-fed and MD-fed baboons (Figures 6C,D).

**Figure 7:**
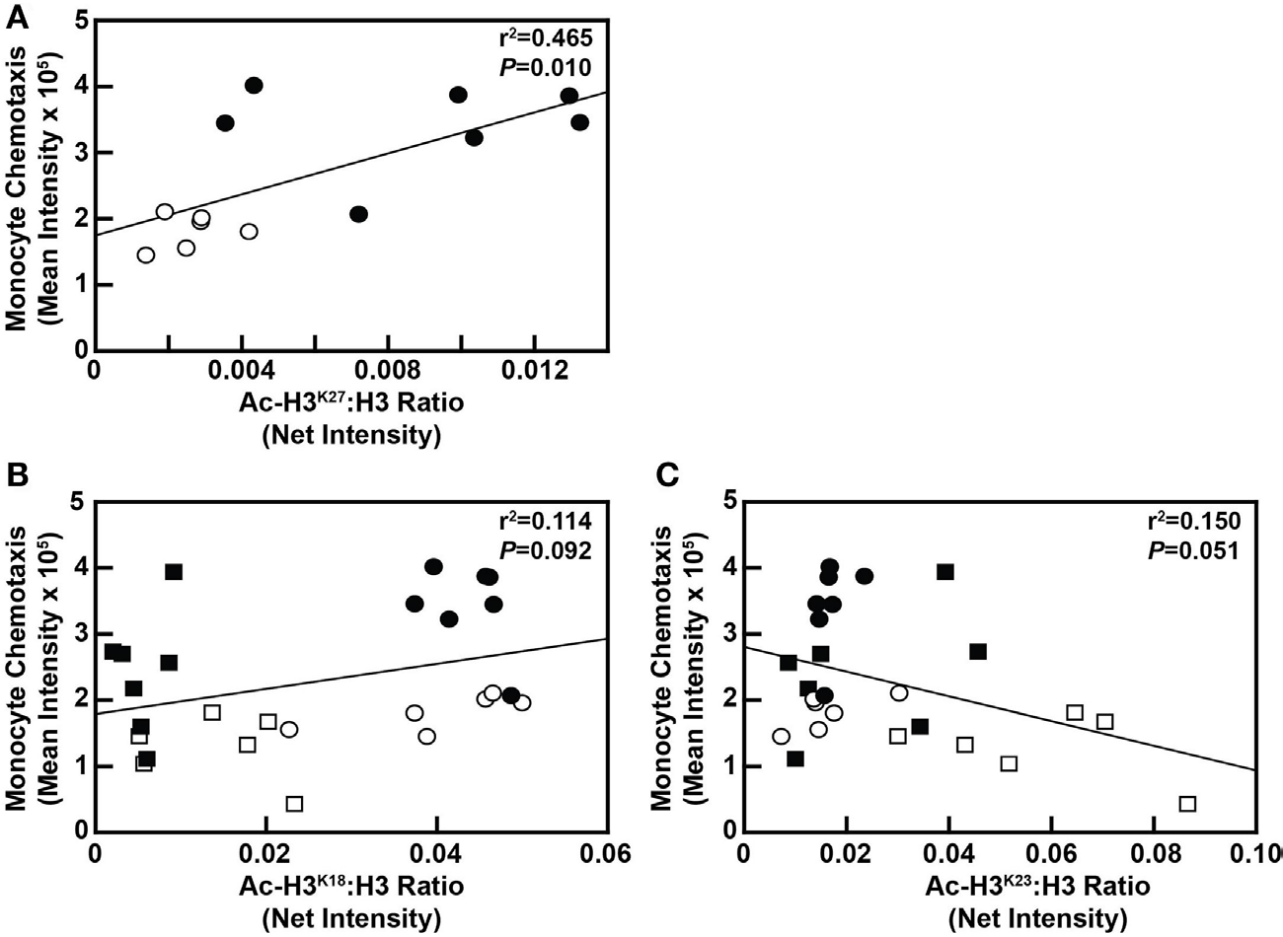
Monocyte chemotaxis is correlated with select H3 acetylation events in maintenance diet (MD)-fed and dyslipidemic diet (DD)-fed baboons. **(A)** Linear regression analysis assessing the relationship between Ac-H3^K27^ levels in monocytes with monocyte chemotaxis from baboons fed an MD (⚪) or a DD (⚫) for 8 weeks. **(B)** Linear regression analysis examining the relationship between Ac-H3^K18^ levels in monocytes with monocyte chemotaxis from baboons fed an MD for 4 weeks (□) or 8 weeks (⚪), or a DD for 4 weeks (■) or 8 weeks (⚫). **(C)** Linear regression analysis examining the relationship between Ac-H3^K23^ levels in monocytes with monocyte chemotaxis from baboons fed an MD for 4 weeks (□) or 8 weeks (⚪), or a DD for 4 weeks (■) or 8 weeks (⚫). In each case, the exact *r*^2^ value and *P-*value for the correlation analysis are shown.

Although blood cholesterol levels and MKP-1 activity in blood monocytes was not found to be correlated with H3 acetylation at any of the aforementioned residues (data not shown), monocyte chemotaxis was significantly correlated with Ac-H3^K27^ at the 8-week time point (*P* = 0.010; Figure 7A). However, monocyte chemotaxis was not significantly correlated with Ac-H3^K27^ at the 4-week time point (*P* = 0.693; data not shown). In addition, the correlation between monocyte chemotaxis and Ac-H3^K18^ status in these cells (*P* = 0.092) as well as Ac-H3^K23^ (*P* = 0.051) nearly reached significance (Figures 7B,C). These data, when taken together, suggest that metabolic priming of blood monocytes caused by a DD is associated with dynamic epigenetic changes in these cells.

## Discussion

The primary goal of this study was to determine whether monocyte priming occurs in NHP in response to a DD, to address potential mechanisms underlying blood monocyte priming and the nature of monocyte dysfunction, and to identify potential epigenetic changes in these metabolically primed monocytes. Our results indicate that purified primary monocytes from baboons, such as monocytes from mice and humans, can be metabolically primed *in vitro*, displaying a sevenfold higher rate of CCL2-induced chemotaxis when cultured under metabolic stress conditions, i.e., high glucose and high LDL (Figure 2A). The degree of priming in baboon monocyte was somewhat surprising since previous studies by our group showed that primed THP-1 monocytes showed only a 1.7-fold to 2-fold increase in CCL2-induced chemotaxis under these same conditions (15, 17, 18). Similarly, human blood monocytes, which were purified by negative selection, displayed a 1.5-fold to 3-fold increases in CCL2-induced chemotaxis (see text footnote 1). One possibility for this discrepancy in *in vitro* monocyte priming is that primary baboon monocytes used in this study were purified by positive selection of classical monocytes (CMs: CD14^hi^). In mice and humans, CMs have been shown to have higher levels of the CCL2 receptor, C-C chemokine receptor type 2 (CCR2) (29), and CMs from rodents were shown to migrate into both inflamed and non-inflamed tissues at a higher rate when compared with non-classical monocytes (NCMs: CD14^lo^CD16^hi^) (29). In addition, a recent study has identified at least two additional human monocyte subsets in addition to CMs and NCMs, which are a heterogeneous cell population within the intermediate monocyte (IM: CD14^hi^:CD16^hi^) subset (30). Whether metabolic stress differentially affects monocyte chemotaxis in different monocyte subsets, i.e., differentially primes monocyte subsets, in response to CCL2 and other chemokines was not investigated as part of this study. Our data also show that *in vivo* metabolic stress generated by a DD in baboons metabolically primes blood monocytes, rendering them hyperresponsive to CCL2 (Figure 2C). These data further support our previous studies showing that metabolic stress induced by feeding mice obesogenic, diabetogenic, or atherogenic diets promotes monocyte hyperresponsiveness to chemokines (13, 14). Interestingly, metabolic priming of baboon monocytes in response to a DD continually increased throughout the 8-week study and had potentially not reached its peak at this time point.

Our data also show that the timing of DD-enhanced monocyte chemotaxis and DD-mediated inhibition of MKP-1 activity are incongruous in baboons during this 8-week study. Furthermore, feeding baboons a DD for up to 8 weeks did not significantly alter p38 or ERK signaling in monocytes, at least at baseline levels. These data do not appear to match previous *in vitro* data from our group demonstrating that metabolic priming of THP-1 monocytes was facilitated by the *S*-glutathionylation and inactivation of the MKP-1 protein, thereby leading to enhanced ERK and p38 MAPK signaling in response to CCL2 (19). However, in this study, we only analyzed the ERK and p38 MAPK activation status in resting monocytes but not in CCL2-stimulated cells. We have also shown that *S*-glutathionylation of β-actin and 14-3-3ζ contribute to monocyte priming *in vitro* (15, 16), indicating that multiple mechanisms contribute to the hyper-chemotactic response of primed monocytes to chemoattractants. Furthermore, we showed that increased protein-*S*-glutathionylation is correlated with monocyte priming *in vivo* (13, 15), and we have recently identified over 130 proteins that are *S*-glutathionylated in monocytes and macrophages, many of which are altered in response to metabolic stress (31). Therefore, it is possible that at least at these early time points, *S*-glutathionylated protein(s) other than MKP-1, e.g., β-actin and 14-3-3ζ, contribute to monocyte priming in baboon monocytes *in vivo*.

Glutaredoxin 1 protein levels did not differ in monocytes from MD-fed and DD-fed baboons. However, we cannot rule out that in response to a DD Grx1 was inactivated. It is also possible that, at least at these early stages, monocyte priming in baboon monocytes *in vivo* is regulated in a manner that is not controlled by protein-*S*-glutathionylation.

In addition to monocyte priming, obesity and CMS have been reported to lead to abnormal hematopoiesis, including increased counts of neutrophils, monocytes, and platelets, which contribute to atherogenesis and the subsequent cardiovascular events resulting from CMS [reviewed in Ref. (32)]. For example, an increase in circulating monocytes, especially NCMs and IMs, has been demonstrated in obese human patients when compared with lean or normal-weight individuals (28, 33). In addition, platelet size, as measured by the mean platelet volume (MPV), is also increased in patients with CMS and is associated with cardiovascular events; however, results linking increased MPV with hypercholesterolemia have been conflicting [reviewed in Ref. (32, 34)]. Our results indicate that in response to a DD, NHPs display a temporal change in hematopoiesis that accompanies hypercholesterolemia, whereby neutrophilic leukocytosis precedes increased platelet counts, and both of these hematopoietic changes precede changes in monocyte counts or altered monocyte subset composition (Figure 3 and data not shown). By contrast, metabolic priming of monocytes occurred as early as neutrophilia and also preceded changes in monocyte levels or changes in monocyte subset composition. Lastly, no significant changes in MPV were observed over the course of this 8-week study (data not shown), indicating that increased MPV occurs later than metabolic priming of monocytes, neutrophilia, and increased platelet counts. It also suggests that increased MPV is driven by factors other than hypercholesterolemia.

Thus far, much of the research concerning hypercholesterolemia-induced monocytosis and neutrophilia has focused on the effects of hypercholesterolemia on hematopoietic stem/progenitor cells (HSPCs). Hypercholesterolemia has multiple effects on HSPCs, including (i) inducing HSPC proliferation and differentiation, (ii) mobilizing HSPCs for release from the bone marrow niche, and (iii) potentiating HSPCs for homing to extramedullary sites, including sites of tissue damage [reviewed in Ref. (35, 36)]. In rodent models of obesity, Nagareddy et al. showed that obesity is associated with both monocytosis and neutrophilia. Both events appear to be driven by adipose tissue macrophages, which secrete IL-1β and promote proliferation of both common myeloid progenitor cells and granulocyte macrophage progenitor cells (37). However, our data showing that neutrophilia precedes monocytosis suggests that there is a disparate mechanism regulating these two hematopoietic events, possibly directly in the bone marrow, at least at early time points. For example, it is possible that early-stage hypercholesterolemia in baboons enhances mobilization of neutrophils from the bone marrow, which is facilitated by CXC chemokine receptor 2, without yet affecting monocyte mobilization, which is facilitated by CCR2 (36).

Lastly, there is increasing evidence that supports interactions between intracellular metabolism and immune function, with epigenetics and histone modifications playing a critical role in regulating immune cell functions and in immunometabolism (38). For example, histone acetyltransferases (HATs) utilize the small intracellular metabolite, acetyl-coenzyme A, as a substrate to acetylate both histone and non-histone proteins in immune cells and in other cell types (39). In addition, monocytes from diabetic patients compared with cells from healthy volunteers show increased acetylation of histone H3 at lysine 9 (Ac-H3^K9^) and lysine 14 (Ac-H3^K14^) in both the tumor necrosis factor α (*TNF-*α) and *PTGS2* (cyclooxygenase 2) promoters (40). Our results indicate that both MD feeding and DD feeding have selective effects on H3 acetylation at various lysine residues over the course of this 8-week study. When compared with standard baboon chow, the MD contained several differences in dietary composition that could account for MD-mediated effects, including a 36% higher cholesterol content and an 8% higher metabolizable energy (kilocalories/gram) content. However, there were also differences in several minerals and vitamins when comparing the MD and standard chow. Nonetheless, our results also indicate that metabolically primed monocytes exhibit altered histone H3 acetylation at three lysine residues at either the 4- or 8-week time point. First, we observed hypoacetylation of H3^K18^ and H3^K23^ at the 4-week time point (Figures 6C,D). Although hypoacetylation of H3 at these two sites in monocytes has not been reported, previous studies have shown histone deacetylase 3 (HDAC3)-mediated H3^K18^ and H3^K23^ hypoacetylation occurs during the mitotic phase of 293 T cells (41). It is unlikely that H3^K18^ and H3^K23^ hypoacetylation is occurring due to hyperproliferation of monocytes since blood monocyte concentrations remained unchanged (Figure 3B). In addition, we observed no changes in phosphorylated H3^S10^, which is an epigenetic mark of cell proliferation (42) and is highly enriched in monocytes compared with macrophages (43). However, it is possible that H3^K18^ and H3^K23^ hypoacetylation poises primed monocytes for hyperproliferation once they extravasate into inflamed tissues. At this point, the underlying molecular mechanisms for H3^K18^ and H3^K23^ hypoacetylation in primed baboon monocytes is unclear, but both increased activity of the HDACs that regulate H3^K18^ and H3^K23^ acetylation, e.g., HDAC3, Sirtuin 7 (Sirt7), or Sirt2 (41, 44, 45), or decreased activity of HATs or transcriptional regulators shown to facilitate the acetylation of H3^K18^, such as E1A binding protein p300 (p300) or CREB-binding protein (46), may have contributed to this effect.

Our data also show that metabolic priming is correlated with Ac-H3^K27^ in monocytes at the 8-week time point, suggesting that primed monocytes will impact the macrophages derived from these altered monocytes. Thus, altered Ac-H3^K27^, or Ac-H3^K18^ or Ac-H3^K23^ for that matter, could impact the differentiation of monocytes into macrophages. Ac-H3^K27^ is acquired on enhancers with active RNA transcription in terminally differentiated hematopoietic cells, including monocytes, macrophages, granulocytes, and pro-erythrocytes (EryB) (47), and differential distribution of Ac-H3^K27^ at active enhancers between monocytes and MDMs is associated with lineage-determining transcription factor binding sites that are important in driving cell differentiation (48–50). Similarly, Wallner et al. recently identified differentially methylated DNA regions (DMRs) around the transcription start site of 4,766 genes that were differentially expressed in differentiated macrophages compared with monocytes, and Ac-H3^K27^ was upregulated in 2 of 3 clusters of these DMRs as a characteristic of active enhancers (51). Changes in Ac-H3^K27^, Ac-H3^K18^, or Ac-H3^K23^Ac-H3 in metabolically primed monocytes could also impact the activation of MDMs. Ac-H3^K27^ is the most dynamically changing histone mark in response to lipopolysaccharide (LPS)-induced “tolerance” and β-glucan-induced “training” in human blood MDMs, changing at 19% of regulatory elements (50). In addition, approximately 65,000 regulatory elements exhibit changes in Ac-H3^K27^ in response to LPS and other macrophage-activating stimuli, and changes in Ac-H3^K27^ at these regulatory elements are associated with transcription factor recruitment in response to macrophage stimulation (52). For example, hyperacetylated enhancers induced by LPS are enriched in activatory protein-1 binding sites, whereas hyperacetylated and signal transducer and activator of transcription-enriched sites are associated with interleukin-4 and interferon-γ (IFNγ) stimulation (52). Thus, our data strongly suggest that dyslipidemia induced by a DD alters the epigenetic landscape in blood monocytes, thereby reprogramming the metabolic memory of these cells and that particularly changes in H3 acetylation may poise primed blood monocytes to differentiate into a hyperproliferative MDM with dysregulated activation states. In support of this hypothesis, we reported that primed MDMs as well as MKP-1-deficient macrophage convert into a hyper-inflammatory phenotype in response to IFNγ + TNFα activation but show a blunted inflammation resolving phenotype in response to interleukin-4 stimulation (19). Further elucidation of how metabolic stress alters epigenetics, especially at specific promoters that control monocyte differentiation and macrophage functions, is needed to improve our understanding of the links between metabolic disorders, the dysregulation of the immune system and the onset and progression of chronic inflammatory diseases, including obesity, cardiovascular diseases, and certain types of cancers.

## Ethics Statement

This study was carried out in accordance with the recommendations of the US Public Health Service Guide for the Care and Use of Laboratory Animals, the US Animal Welfare Act, and the Institutional Animal Care and Use Committee at the Texas Biomedical Research Institute (TBRI). The protocol was approved by the TBRI Institutional Animal Care and Use Committee and conducted in Association for Assessment and Accreditation of Laboratory Animal Care-approved facilities.

## Author Contributions

This study was designed by JS, RA, and LC with RA as the principal investigator. The study was directed and coordinated by JS and RA with LC provided conceptual and technical guidance for aspects of the study related to NHPs. JS and ST designed and optimized the monocyte purification strategy. The monocyte chemotaxis assays and MKP-1 activity assays were designed and executed by HN and AC with oversight from JS. Western blotting assays were performed by CF, AC, and JS. The manuscript was written by JS and RA; comments and edits were provided by all other authors (ST, HN, AC, CF, and LC).

## Conflict of Interest Statement

The authors declare that the research was conducted in the absence of any commercial or financial relationships that could be construed as a potential conflict of interest.

## Footnotes

^1^Nguyen HN, Farnen CM, Ullevig SL, Short JD, Asmis R. Structure-function analysis of ursolic acid analogues identifies 23-hydroxy ursolic acid as a potential novel inhibitor of monocyte dysfunction associated with metabolic disorders. *Phytochemistry* (2017) (submitted).

## Supplementary Material

Figure S1 Baboon blood monocyte analysis and purification. **(A)** Gating strategy for monocyte subset analysis. Blood leukocytes, which were stained with PE-conjugated CD11b, APC-conjugated CD14, and AF488-conjugated CD16 antibodies, were gated as shown and analyzed by flow cytometry. **(B)** Flow cytometry analysis of leukocytes following purification of baboon blood monocytes, as described in Section “Materials and Methods.” The monocyte population is outlined by the quadrilateral polygon, and the monocyte percentage within the polygon is indicated.

Figure S2 DD feeding does not alter baseline p38 or extracellular signal-regulated kinase (ERK) signaling in baboon monocytes. **(A)** Western blot analysis of p38 and ERK signaling pathways using lysates from monocytes, which were purified from baboons that were fed an MD or a DD for either 4 weeks (left panels) or 8 weeks (right panels). **(B)** The densitometric ratio (mean ± SEM) of p-p38^T180/Y182^: p38, which was calculated using images shown in panel **(A)**. **(C)** The densitometric ratio (mean ± SEM) of p-ERK^T202/Y204^: ERK, which was calculated using images shown in panel **(A)**. NS, not statistically significant.

Figure S3 DD feeding leads to reduced histone H3 acetylation at lysine 18 and 23 in baboon monocytes. Western blot analysis of acetylated H3 at the indicated residues, or total H3, using lysates generated from MD-fed or DD-fed baboon monocytes—monocytes were purified from baboons at either the 4-week time point (left panels) or 8-week time point (right panels).

## References

[1] Beltran-Sanchez H Harhay MO Harhay MM McElligott S. Prevalence and trends of metabolic syndrome in the adult U.S. population, 1999-2010. J Am Coll Cardiol (2013) 62:697–703. 10.1016/j.jacc.2013.05.064 23810877 PMC3756561

[2] Kelli HM Kassas I Lattouf OM Cardio metabolic syndrome: a global epidemic. Diabetes Metab (2015) 6:1–14. 10.4172/2155-6156.1000513

[3] von Bibra H Paulus W St John Sutton M. Cardiometabolic syndrome and increased risk of heart failure. Curr Heart Fail Rep (2016) 13:219–29. 10.1007/s11897-016-0298-4 27539049 PMC5069335

[4] Esser N Legrand-Poels S Piette J Scheen AJ Paquot N. Inflammation as a link between obesity, metabolic syndrome and type 2 diabetes. Diabetes Res Clin Pract (2014) 105:141–50. 10.1016/j.diabres.2014.04.006 24798950

[5] O’Rourke RW White AE Metcalf MD Olivas AS Mitra P Larison WG Hypoxia-induced inflammatory cytokine secretion in human adipose tissue stromovascular cells. Diabetologia (2011) 54:1480–90. 10.1007/s00125-011-2103-y 21400042 PMC3159546

[6] Weisberg SP McCann D Desai M Rosenbaum M Leibel RL Ferrante AW Jr. Obesity is associated with macrophage accumulation in adipose tissue. J Clin Invest (2003) 112:1796–808. 10.1172/JCI19246 14679176 PMC296995

[7] Short JD Downs K Tavakoli S Asmis R. Protein thiol redox signaling in monocytes and macrophages. Antioxid Redox Signal (2016) 25:816–35. 10.1089/ars.2016.6697 27288099 PMC5107717

[8] Tavakoli S Asmis R. Reactive oxygen species and thiol redox signaling in the macrophage biology of atherosclerosis. Antioxid Redox Signal (2012) 17:1785–95. 10.1089/ars.2012.4638 22540532 PMC3474194

[9] Kennedy AJ Ellacott KL King VL Hasty AH. Mouse models of the metabolic syndrome. Dis Model Mech (2010) 3:156–66. 10.1242/dmm.003467 20212084 PMC2869491

[10] Ishibashi S Brown MS Goldstein JL Gerard RD Hammer RE Herz J. Hypercholesterolemia in low density lipoprotein receptor knockout mice and its reversal by adenovirus-mediated gene delivery. J Clin Invest (1993) 92:883–93. 10.1172/JCI116663 8349823 PMC294927

[11] Merat S Casanada F Sutphin M Palinski W Reaven PD Western-type diets induce insulin resistance and hyperinsulinemia in LDL receptor-deficient mice but do not increase aortic atherosclerosis compared with normoinsulinemic mice in which similar plasma cholesterol levels are achieved by a fructose-rich diet. Arterioscler Thromb Vasc Biol (1999) 19:1223–30. 10.1161/01.ATV.19.5.1223 10323773

[12] Towler DA Bidder M Latifi T Coleman T Semenkovich CF. Diet-induced diabetes activates an osteogenic gene regulatory program in the aortas of low density lipoprotein receptor-deficient mice. J Biol Chem (1998) 273:30427–34. 10.1074/jbc.273.46.30427 9804809

[13] Qiao M Zhao Q Lee CF Tannock LR Smart EJ LeBaron RG Thiol oxidative stress induced by metabolic disorders amplifies macrophage chemotactic responses and accelerates atherogenesis and kidney injury in LDL receptor-deficient mice. Arterioscler Thromb Vasc Biol (2009) 29:1779–86. 10.1161/ATVBAHA.109.191759 19592463 PMC2766026

[14] Ullevig SL Zhao Q Zamora D Asmis R. Ursolic acid protects diabetic mice against monocyte dysfunction and accelerated atherosclerosis. Atherosclerosis (2011) 219:409–16. 10.1016/j.atherosclerosis.2011.06.013 21752377 PMC3199329

[15] Ullevig S Zhao Q Lee CF Seok Kim H Zamora D Asmis R. NADPH oxidase 4 mediates monocyte priming and accelerated chemotaxis induced by metabolic stress. Arterioscler Thromb Vasc Biol (2012) 32:415–26. 10.1161/ATVBAHA.111.238899 22095986 PMC3262086

[16] Kim HS Ullevig SL Nguyen HN Vanegas D Asmis R Redox regulation of 14-3-3zeta controls monocyte migration. Arterioscler Thromb Vasc Biol (2014) 34:1514–21. 10.1161/ATVBAHA.114.303746 24812321 PMC4065841

[17] Kim HS Ullevig SL Zamora D Lee CF Asmis R. Redox regulation of MAPK phosphatase 1 controls monocyte migration and macrophage recruitment. Proc Natl Acad Sci U S A (2012) 109:E2803–12. 10.1073/pnas.1212596109 22991462 PMC3478659

[18] Ullevig SL Kim HS Nguyen HN Hambright WS Robles AJ Tavakoli S Ursolic acid protects monocytes against metabolic stress-induced priming and dysfunction by preventing the induction of Nox4. Redox Biol (2014) 2:259–66. 10.1016/j.redox.2014.01.003 24494201 PMC3909821

[19] Kim HS Tavakoli S Piefer LA Nguyen HN Asmis R. Monocytic MKP-1 is a sensor of the metabolic environment and regulates function and phenotypic fate of monocyte-derived macrophages in atherosclerosis. Sci Rep (2016) 6:34223. 10.1038/srep34223 27670844 PMC5037453

[20] Tejero ME Rodriguez-Sanchez IP Barrera-Saldana HA In: Barrera Saldaña HA, editor. Monkeys: Brain Development, Social & Hormonal Mechanisms and Zoonotic Diseases. (Chap. 10), Hauppauge, NY: Nova Science Publishers, Inc (2014). p. 203–22.

[21] Kritchevsky D Davidson LM Shapiro IL Kim HK Kitagawa M Malhotra S Lipid metabolism and experimental atherosclerosis in baboons: influence of cholesterol-free, semi-synthetic diets. Am J Clin Nutr (1974) 27:29–50. 4358391 10.1093/ajcn/27.1.29

[22] Strong JP McGill HC Jr Diet and experimental atherosclerosis in baboons. Am J Pathol (1967) 50:669–90. 4960563 PMC1965309

[23] Rainwater D VandeBerg JL The Baboon in Biomedical Research. New York, NY: Springer (2009). p. 225–36.

[24] Higgins PB Bastarrachea RA Lopez-Alvarenga JC Garcia-Forey M Proffitt JM Voruganti VS Eight week exposure to a high sugar high fat diet results in adiposity gain and alterations in metabolic biomarkers in baboons (*Papio hamadryas* sp.). Cardiovasc Diabetol (2010) 9:71. 10.1186/1475-2840-9-71 21034486 PMC2988722

[25] MacCluer JW Kammerer CM Blangero J Dyke B Mott GE VandeBerg JL Pedigree analysis of HDL cholesterol concentration in baboons on two diets. Am J Hum Genet (1988) 43:401–13. 3177383 PMC1715506

[26] Wintergerst ES Jelk J Asmis R Differential expression of CD14, CD36 and the LDL receptor on human monocyte-derived macrophages. A novel cell culture system to study macrophage differentiation and heterogeneity. Histochem Cell Biol (1998) 110:231–41. 10.1007/s004180050285 9749957

[27] Chang FM Reyna SM Granados JC Wei SJ Innis-Whitehouse W Maffi SK Inhibition of neddylation represses lipopolysaccharide-induced proinflammatory cytokine production in macrophage cells. J Biol Chem (2012) 287:35756–67. 10.1074/jbc.M112.397703 22927439 PMC3471689

[28] Poitou C Dalmas E Renovato M Benhamo V Hajduch F Abdennour M CD14dimCD16+ and CD14+CD16+ monocytes in obesity and during weight loss: relationships with fat mass and subclinical atherosclerosis. Arterioscler Thromb Vasc Biol (2011) 31:2322–30. 10.1161/ATVBAHA.111.230979 21799175

[29] Geissmann F Jung S Littman DR. Blood monocytes consist of two principal subsets with distinct migratory properties. Immunity (2003) 19:71–82. 10.1016/S1074-7613(03)00174-2 12871640

[30] Villani AC Satija R Reynolds G Sarkizova S Shekhar K Fletcher J Single-cell RNA-seq reveals new types of human blood dendritic cells, monocytes, and progenitors. Science (2017) 356:eaah4573. 10.1126/science.aah4573 28428369 PMC5775029

[31] Ullevig SL Kim HS Short JD Tavakoli S Weintraub ST Downs K Protein S-glutathionylation mediates macrophage responses to metabolic cues from the extracellular environment. Antioxid Redox Signal (2016) 25:836–51. 10.1089/ars.2015.6531 26984580 PMC5107721

[32] Murphy AJ Tall AR. Disordered haematopoiesis and athero-thrombosis. Eur Heart J (2016) 37:1113–21. 10.1093/eurheartj/ehv718 26869607 PMC4823636

[33] Devêvre EF Renovato-Martins M Clément K Sautès-Fridman C Cremer I Poitou C. Profiling of the three circulating monocyte subpopulations in human obesity. J Immunol (2015) 194:3917–23. 10.4049/jimmunol.1402655 25786686

[34] Vizioli L Muscari S Muscari A The relationship of mean platelet volume with the risk and prognosis of cardiovascular diseases. Int J Clin Pract (2009) 63:1509–15. 10.1111/j.1742-1241.2009.02070.x 19769707

[35] Ma X Feng Y. Hypercholesterolemia tunes hematopoietic stem/progenitor cells for inflammation and atherosclerosis. Int J Mol Sci (2016) 17:E1162. 10.3390/ijms17071162 27447612 PMC4964534

[36] Soehnlein O Swirski FK. Hypercholesterolemia links hematopoiesis with atherosclerosis. Trends Endocrinol Metab (2013) 24:129–36. 10.1016/j.tem.2012.10.008 23228326 PMC4302393

[37] Nagareddy PR Kraakman M Masters SL Stirzaker RA Gorman DJ Grant RW Adipose tissue macrophages promote myelopoiesis and monocytosis in obesity. Cell Metab (2014) 19:821–35. 10.1016/j.cmet.2014.03.029 24807222 PMC4048939

[38] Raghuraman S Donkin I Versteyhe S Barres R Simar D. The emerging role of epigenetics in inflammation and immunometabolism. Trends Endocrinol Metab (2016) 27:782–95. 10.1016/j.tem.2016.06.008 27444065

[39] Kaochar S Tu BP. Gatekeepers of chromatin: small metabolites elicit big changes in gene expression. Trends Biochem Sci (2012) 37:477–83. 10.1016/j.tibs.2012.07.008 22944281 PMC3482309

[40] Miao F Gonzalo IG Lanting L Natarajan R. In vivo chromatin remodeling events leading to inflammatory gene transcription under diabetic conditions. J Biol Chem (2004) 279:18091–7. 10.1074/jbc.M311786200 14976218

[41] Li Y Kao GD Garcia BA Shabanowitz J Hunt DF Qin J A novel histone deacetylase pathway regulates mitosis by modulating aurora B kinase activity. Genes Dev (2006) 20:2566–79. 10.1101/gad.1455006 16980585 PMC1578679

[42] Nowak SJ Corces VG. Phosphorylation of histone H3: a balancing act between chromosome condensation and transcriptional activation. Trends Genet (2004) 20:214–20. 10.1016/j.tig.2004.02.007 15041176

[43] Nicholas D Tang H Zhang Q Rudra J Xu F Langridge W Quantitative proteomics reveals a role for epigenetic reprogramming during human monocyte differentiation. Mol Cell Proteomics (2015) 14:15–29. 10.1074/mcp.M113.035089 25316709 PMC4288251

[44] Barber MF Michishita-Kioi E Xi Y Tasselli L Kioi M Moqtaderi Z SIRT7 links H3K18 deacetylation to maintenance of oncogenic transformation. Nature (2012) 487:114–8. 10.1038/nature11043 22722849 PMC3412143

[45] Eskandarian HA Impens F Nahori MA Soubigou G Coppée JY Cossart P A role for SIRT2-dependent histone H3K18 deacetylation in bacterial infection. Science (2013) 341:1238858. 10.1126/science.1238858 23908241

[46] Horwitz GA Zhang K McBrian MA Grunstein M Kurdistani SK Berk AJ. Adenovirus small e1a alters global patterns of histone modification. Science (2008) 321:1084–5. 10.1126/science.1155544 18719283 PMC2756290

[47] Lara-Astiaso D Weiner A Lorenzo-Vivas E Zaretsky I Jaitin DA David E Immunogenetics. Chromatin state dynamics during blood formation. Science (2014) 345:943–9. 10.1126/science.1256271 25103404 PMC4412442

[48] Heinz S Romanoski CE Benner C Allison KA Kaikkonen MU Orozco LD Effect of natural genetic variation on enhancer selection and function. Nature (2013) 503:487–92. 10.1038/nature12615 24121437 PMC3994126

[49] Pham TH Benner C Lichtinger M Schwarzfischer L Hu Y Andreesen R Dynamic epigenetic enhancer signatures reveal key transcription factors associated with monocytic differentiation states. Blood (2012) 119:e161–71. 10.1182/blood-2012-01-402453 22550342

[50] Saeed S Quintin J Kerstens HH Rao NA Aghajanirefah A Matarese F Epigenetic programming of monocyte-to-macrophage differentiation and trained innate immunity. Science (2014) 345:1251086. 10.1126/science.1251086 25258085 PMC4242194

[51] Wallner S Schröder C Leitão E Berulava T Haak C Beißer D Epigenetic dynamics of monocyte-to-macrophage differentiation. Epigenetics Chromatin (2016) 9:33. 10.1186/s13072-016-0079-z 27478504 PMC4967341

[52] Ostuni R Piccolo V Barozzi I Polletti S Termanini A Bonifacio S Latent enhancers activated by stimulation in differentiated cells. Cell (2013) 152:157–71. 10.1016/j.cell.2012.12.018 23332752

