# Supplementary material for: Dyslipidemic Diet-Induced Monocyte “Priming” and Dysfunction in Non-Human Primates Is Triggered by Elevated Plasma Cholesterol and Accompanied by Altered Histone Acetylation
Source: Front Immunol. 2017 Aug 22;8:958. doi: 10.3389/fimmu.2017.00958 (PMC5572238; doi:10.3389/fimmu.2017.00958)
Supplement: Supplementary file 1 [file Table_1.docx]

**Supplementary Table 1. Monocyte subset composition (mean ± SEM)**

|  | **4 Weeks** | | |
| --- | --- | --- | --- |
| **Monocyte Subset** | **MD (n=6)** | **HFD (n=7)** | ***P*-Value** |
| Non-classical monocytes (CD14^lo^CD16^hi^) | 2.25 ± 0.31 | 2.34 ± 0.34 | 0.85 |
| Intermediate monocytes (CD14^hi^CD16^hi^) | 10.65 ± 1.97 | 7.16 ± 0.91 | 0.12 |
| Classical monocytes (CD14^hi^CD16^lo^) | 72.87 ± 2.15 | 66.59 ± 3.98 | 0.21 |
|  | **8 Weeks** | | |
| **Monocyte Subset** | **MD (n=6)** | **HFD (n=7)** | ***P*-Value** |
| Non-classical monocytes (CD14^lo^CD16^hi^) | 3.50 ± 0.62 | 4.01 ± 0.81 | 0.63 |
| Intermediate monocytes (CD14^hi^CD16^hi^) | 5.36 ± 0.83 | 4.50 ± 0.52 | 0.45 |
| Classical monocytes (CD14^hi^CD16^lo^) | 52.58 ± 2.65 | 52.21 ± 3.02 | 0.93 |
