# Supplementary figures and images for: Dyslipidemic Diet-Induced Monocyte “Priming” and Dysfunction in Non-Human Primates Is Triggered by Elevated Plasma Cholesterol and Accompanied by Altered Histone Acetylation
Source: Front Immunol. 2017 Aug 22;8:958. doi: 10.3389/fimmu.2017.00958 (PMC5572238; doi:10.3389/fimmu.2017.00958)

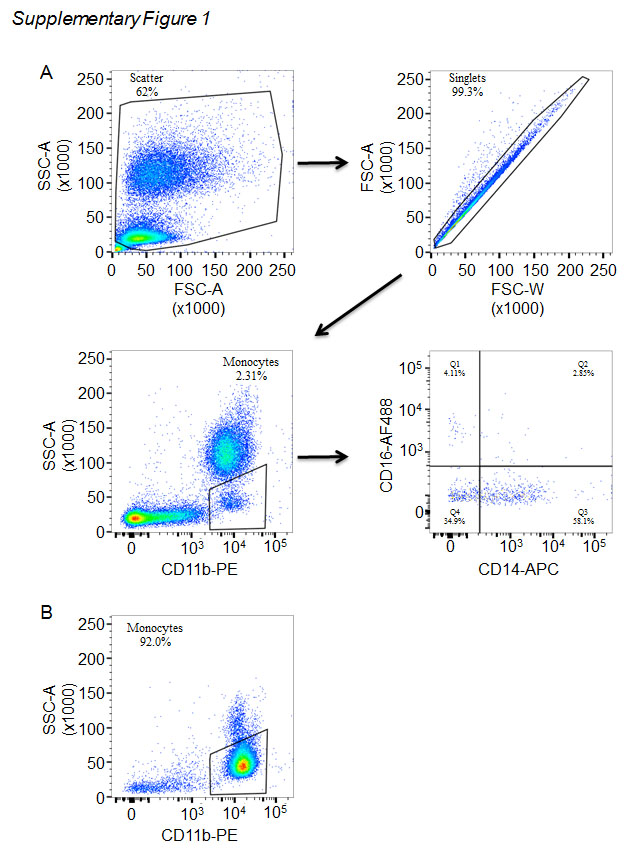

Supplement: Figure S1 — Baboon blood monocyte analysis and purification. (A) Gating strategy for monocyte subset analysis. Blood leukocytes, which were stained with PE-conjugated CD11b, APC-conjugated CD14, and AF488-conjugated CD16 antibodies, were gated as shown and analyzed by flow cytometry. (B) Flow cytometry analysis of leukocytes following purification of baboon blood monocytes, as described in Section “Materials and Methods.” The monocyte population is outlined by the quadrilateral polygon, and the monocyte percentage within the polygon is indicated. [file Image_1.JPEG]

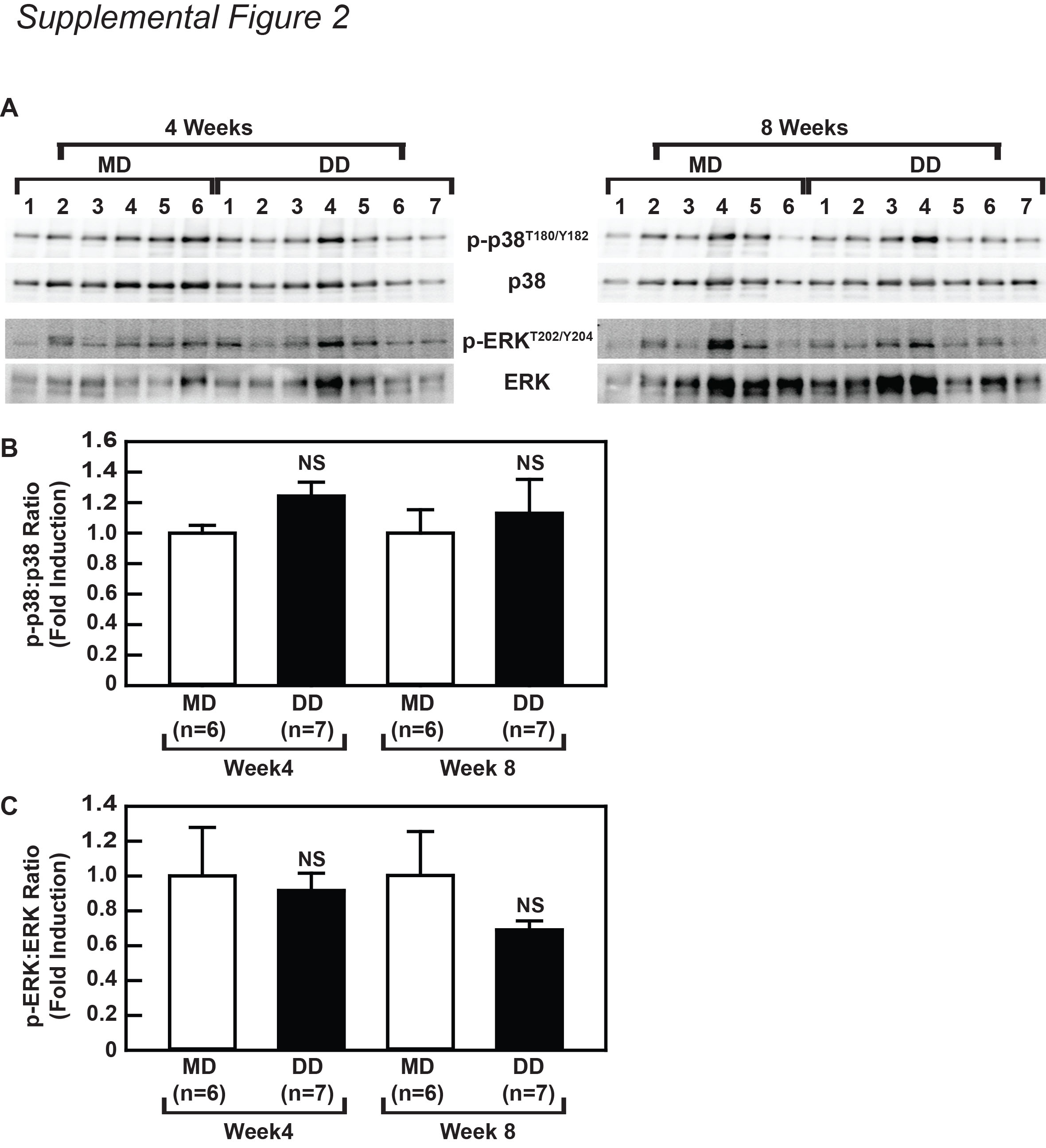

Supplement: Figure S2 — DD feeding does not alter baseline p38 or extracellular signal-regulated kinase (ERK) signaling in baboon monocytes. (A) Western blot analysis of p38 and ERK signaling pathways using lysates from monocytes, which were purified from baboons that were fed an MD or a DD for either 4 weeks (left panels) or 8 weeks (right panels). (B) The densitometric ratio (mean ± SEM) of p-p38T180/Y182: p38, which was calculated using images shown in panel (A). (C) The densitometric ratio (mean ± SEM) of p-ERKT202/Y204: ERK, which was calculated using images shown in panel (A). NS, not statistically significant. [file Image_2.JPEG]

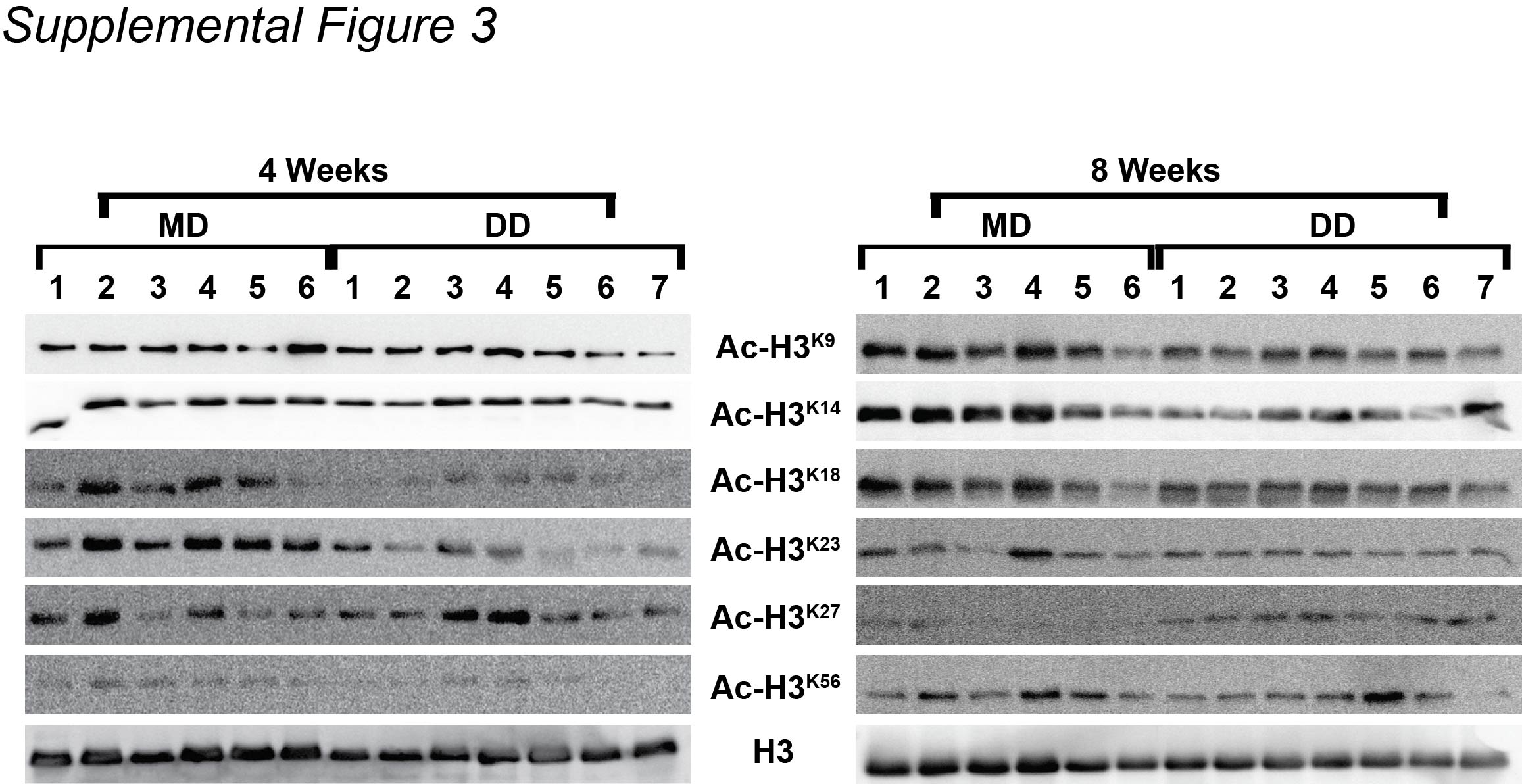

Supplement: Figure S3 — DD feeding leads to reduced histone H3 acetylation at lysine 18 and 23 in baboon monocytes. Western blot analysis of acetylated H3 at the indicated residues, or total H3, using lysates generated from MD-fed or DD-fed baboon monocytes—monocytes were purified from baboons at either the 4-week time point (left panels) or 8-week time point (right panels). [file Image_3.JPEG]
